# THE LONELINESS OF LOW-INCOME OLDER ADULTS IN A FEDERAL VOLUNTEERING PROGRAM: A MIXED-METHOD NETWORK PERSPECTIVE

**DOI:** 10.1093/geroni/igad104.0072

**Published:** 2023-12-21

**Authors:** Katy (Qiuchang) Cao

**Affiliations:** Florida State University, Tallahassee, Florida, United States

## Abstract

Volunteering is considered an important way to prevent and address loneliness for community-living older adults by fostering social interactions. However, some social network studies conducted outside of the volunteering context suggest that increased interactions among lonely individuals can elevate network members’ loneliness through the co-rumination of negative emotions. To understand how and why interactions among older volunteers might protect against loneliness, this study conducted a concurrent mixed-method social network analysis among older volunteers within the Senior Companion Program (SCP) in Columbus, Ohio. These volunteers are 55 years or older, at or below 200% of the federal poverty line, and culturally/linguistically diverse. Qualitative and quantitative data were collected using focus groups and surveys respectively during an SCP monthly in-service training, N=41. A linear network autocorrelation model (LNAM) was constructed to quantify how individuals’ loneliness was correlated in SCP. A grounded theory approach informed the qualitative analysis. Mixed-method integration was conducted through a joint display table. LNAM results on the covariates were consistent with previous loneliness studies on diverse older adults. Furthermore, the negative network autocorrelation indicated that less lonely volunteers tended to interact with lonelier volunteers (ρ= -0.06, p< 0.05). Qualitative results also supported the aforementioned socialization pattern suggestive of altruistic motivations. In other words, the altruistic tendency to interact with other volunteers differing in loneliness may be an important pathway by which volunteering addresses loneliness. Future research shall further examine the mechanism between volunteering and older adults’ psychosocial well-being. Findings have implications for maximizing the health and social benefits of volunteering.

